# Improved delineation model of a standard 12-lead electrocardiogram based on a deep learning algorithm

**DOI:** 10.1186/s12911-023-02233-0

**Published:** 2023-07-28

**Authors:** Annisa Darmawahyuni, Siti Nurmaini, Muhammad Naufal Rachmatullah, Prazna Paramitha Avi, Samuel Benedict Putra Teguh, Ade Iriani Sapitri, Bambang Tutuko, Firdaus Firdaus

**Affiliations:** Intelligent System Research Group, Faculty of Computer Science, Universitas Sriwijaya, Palembang, 30139 Indonesia

**Keywords:** 12-lead electrocardiogram, Bidirectional long short-term memory, Convolutional neural network, Delineation model, ECG waveform, Isoelectric line

## Abstract

**Background:**

Signal delineation of a standard 12-lead electrocardiogram (ECG) is a decisive step for retrieving complete information and extracting signal characteristics for each lead in cardiology clinical practice. However, it is arduous to manually assess the leads, as a variety of signal morphological variations in each lead have potential defects in recording, noise, or irregular heart rhythm/beat.

**Method:**

A computer-aided deep-learning algorithm is considered a state-of-the-art delineation model to classify ECG waveform and boundary in terms of the P-wave, QRS-complex, and T-wave and indicated the satisfactory result. This study implemented convolution layers as a part of convolutional neural networks for automated feature extraction and bidirectional long short-term memory as a classifier. For beat segmentation, we have experimented beat-based and patient-based approach.

**Results:**

The empirical results using both beat segmentation approaches, with a total of 14,588 beats were showed that our proposed model performed excellently well. All performance metrics above 95% and 93%, for beat-based and patient-based segmentation, respectively.

**Conclusions:**

This is a significant step towards the clinical pertinency of automated 12-lead ECG delineation using deep learning.

## Introduction

The assessment of electrocardiogram (ECG) signals’ waveform morphology is a crucial step designed to assist cardiologists in diagnosing heart diseases [1]. The diverse morphology of heart diseases is becoming more complicated, making the construction of an automated delineation algorithm challenging [2, 3]. ECG delineation plays a vital role in providing amplitudes, ranges, duration, and morphology [4], and it aims to determine the location of the peaks and boundaries (onset and offset) of three main waveforms (i.e., P-wave, QRS-complex, and T-wave) [2].

ECG signals are low bioelectrical signals and are very changeable; therefore, some noises may influence the characteristics of the signals, such as baseline wander, motion artifact, and muscle artifact [2, 5]. This makes ECG delineation through visual examination more difficult for cardiologists [6]. Many works in the literature have explored ECG delineation algorithms based on machine learning and digital signal processing [7–13]. However, a major limitation is that most previous studies used only a single-lead or several leads to generate the delineation algorithm. Single-lead ECG is commonly used for basic heart monitoring, irregular rhythm checks, or observation of the effects of exercise on the ECG [14]. Hence, single-lead ECG monitoring may less accurately measure cardiac electrical activity [15]. In addition, another limitation of using single-lead ECG is a lack of sufficient validation data in actual clinical practice [16]. In our previous study [3, 5, 17], we proposed a state-of-the-art deep learning (DL) algorithm to classify P-wave, QRS-complex, T-wave, and other straight lines or no deflection of ECG (isoelectric line) by using a single-lead, and the study produced outstanding performance (above 98% accuracy and precision). However, the model could not be properly implemented—as specific diagnose, such as myocardial infarction that will show significant ST segment elevation, is mandatory established by examining the number of leads (12-lead ECG) to observe morphological changes, accurate diagnosis and prompt therapeutic measures [18].

In realistic clinical settings, 12-lead ECG, including six limb leads (I, II, III, aVF, aVR, and aVL) and six chest leads (V1, V2, V3, V4, V5, and V6), is a standard test performed in primary and intensive care units and can provide more valuable information than single-lead ECG [19]. The 12-lead ECG is a practical and cost-effective strategic alternative to routine echocardiography [20]. Some heart diseases require a standard 12-lead ECG observation because each ECG signal has a different heart vector orientation.

Automated computer analysis of standard 12-lead ECG has gained significance in the medical diagnosis process and is becoming more prevalent growing [21]. However, the use of a delineation algorithm for 12-lead ECG is still largely unexplored. Conventional algorithms based on wavelet transform have been implemented for P-wave, QRS-complex, and T-wave detection in 12-lead ECG [22]. However, the algorithm lacks feature analysis and has a high degree of uncertainty due to the subjective measurement aspect. Machine learning (ML) approach, which is a subset of DL has also proposed to waveform delineation in 12-lead ECG [23, 24]. Unfortunately, ML typically requires more ongoing human intervention to feature representation. Whereas with ML systems, we need to identify the applied features based on the type of data, and DL system learns the features without additional human intervention. The model of DL uses distinct layers to learn and discover high-level features from the data on its own. In addition, the delineation of 12-lead ECG is challenging because the resultant ECG pattern may vary when the location of the electrodes on the chest wall is varied. Further, the 12-lead ECG delineation may not be able to detect the boundary of ECG waveforms, as each of the 12-lead ECG represents varying morphology and a different direction of cardiac activation in a three-dimension shape.

To enhance the drawbacks, this study aimed to explore an algorithm for delineating 12-lead ECG using an automated feature extraction method—DL. In our previous study [3], the DL has obtained the outstanding results with the performance above 98% accuracy and precision for single-lead ECG beat delineation. Therefore, in the current study, we have improved and adjusted the previous model to 12-lead ECG delineation. The DL architecture consists of a convolutional neural network (CNN) for feature extraction and bidirectional long short-term memory (bidirectional LSTM/BiLSTM) as a classifier for 12-lead ECG. BiLSTM can be learned to use all available input data for a specific timeframe in the past and future. Both techniques can enhance the ECG waveform classification performance to automatically extract features from the input signals in 12-lead ECG. To the best of our knowledge, we are the first to implement and explore automatically high-level feature representation using DL to delineate 12-lead ECG. Our goal was to improve the delineation model with automated feature representation to 12-lead ECG. For this paper, the main contributions were as follows:

1. Proposing a 12-lead ECG delineation model using CNN for feature extraction and BiLSTM as a classifier. The convolution layer of CNN can generate local features of the ECG signal sequence to recognize regional patterns in the convolution window, and BiLSTM appropriates for sequential data processing based on forward and backward time steps;
2. Classifying ECG waveforms (i.e., P-wave, QRS-complex, T-wave, and isoelectric line) in two scenarios using beat-based and patient-based for 200 recordings (patients) with total 14,588 beats;
3. Training, validating and testing the ECG waveforms by lead-to-lead with total of 12-lead ECG using beat-based and patient-based segmentation approach.

## Materials and methods

### 12-lead ECG

The 12-lead ECG is a standard non-invasive test in cardiology clinical practice [25]. The 12-lead ECG represents the recorded electrical activity of the heart from 10 electrodes on the body surface. An electrode is a conductive pad that is attached to the skin to record electrical activity, which is placed on different parts of limb and chest of patient. The 12-lead ECG consists of six limb leads (I, II, III, aVR, aVL, and aVF) and six chest leads (V1, V2, V3, V4, V5, and V6) [26]. Each of the 12 leads graphically describes the electrical activity of the heart. It represents a particular orientation in space (right arm, RA; left arm, LA; left foot, LL). Table 1 lists the 12-lead ECG electrical activity based on the anatomical relations view.

**Table 1:** The 12-lead ECG electrical activity [27, 28]

Lead	Electrical activity	Anatomical relations view
I	LA-RA	Lateral surface
II	LL-RA	Inferior surface of heart. P-wave mostly clear, and commonly used to observe the rhythm strip.
III	LL-LA	Inferior surface of heart
aVR	RA-average of (LA + LL)	Right atrium and left ventricle cavity
aVL	LA-average of (RA + LL)	Lateral surface
aVF	LL-average of (LA + RA)	Inferior surface of heart
V_1_	V_1_ - average of (LA + RA + LL)	Anterior surface, right atrium and left ventricle cavity
V_2_	V_2_ - average of (LA + RA + LL)	Anterior surface
V_3_	V_3_ - average of (LA + RA + LL)	Anterior surface
V_4_	V_4_ - average of (LA + RA + LL)	Anterior surface
V_5_	V_5_ - average of (LA + RA + LL)	Lateral surface
V_6_	V_6_ - average of (LA + RA + LL)	Lateral surface

### Data preparation

**Fig. 1:**
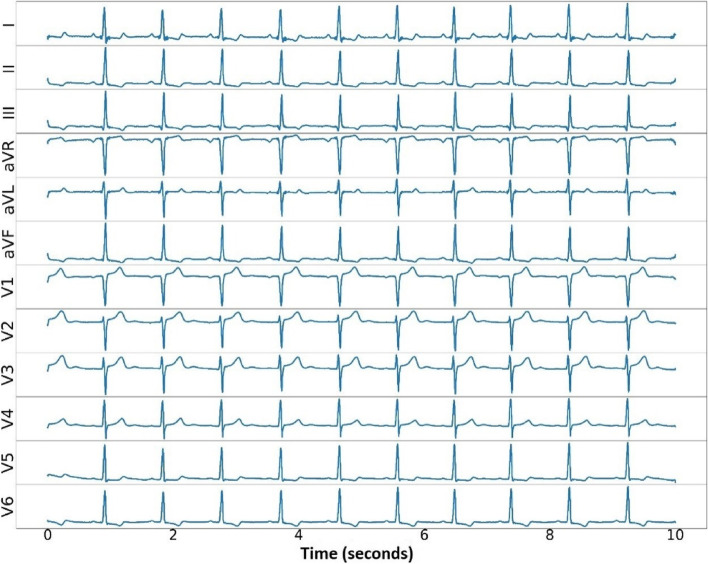
Samples of sinus rhythm in 12-lead ECG signals of LUDB

The proposed algorithm was evaluated on the Lobachevsky University database (LUDB) of ECG signals, which contained 200 records from 200 subjects (healthy volunteers and patients with various heart abnormalities) [29, 30]. The LUDB had a 10-s 12-lead ECG (I, II, III, aVR, aVL, aVF, V1, V2, V3, V4, V5, and V6) digitized at 500 samples per second. The total of 58,429 annotated waves consisted of 16,797 P-waves, 21,966 QRS-complexes, and 19,666 T-waves. The boundaries and peaks of the ECG waveform were manually annotated by two certified and practicing cardiologists. The sample plot of 12-lead ECG signals is presented in Fig. 1.

### Noise removal

Changes in ECG waveforms indicate a cardiac illness that may occur for any reason. ECG signals are enhanced by eliminating various kinds of noise and artifacts. This study proposed discrete wavelet transform, which is a frequently used denoising technique that offers a valuable option for denoising ECG signals [31, 32]. Some wavelet families for ECG signal, such as *symlets* (sym), *daubechies* (db), and *bior*, were implemented to identify and analyze the type of wavelet that will obtain the best signal denoising result. Increasing the signal-to-noise ratio (SNR) to train the DL model is critical. A higher SNR implies better and more trustworthy ECG data. Based on the highest SNR results, bior wavelet or *Bior6.8*, was the best wavelet function and was chosen for ECG signal denoising. The SNR value obtained was 8.44 dB.

### Segmentation

The ECG waveforms were segmented beat to beat in simple way, the process is cutting the ECG signal based on the window size and sampling frequency. For each segmented time window, it contains one heartbeat and has a length of 512 nodes. Each window size has a start of P-wave_1_ to the start of P-wave_2_. If one heartbeat is less than 512 nodes, we perform zero padding technique by adding the value 0 (zero) until the signal has a length of 512 nodes. The LUDB provided the label annotation (ground truth) as waveform onset “(” and offset “)” annotations for P-wave, QRS-complex, and T-wave, respectively. If the annotation doesn’t provide the label onset “(” and offset “)”, the beats segmentation are excluded. A total of 58,429 annotated waves were selected to generate the DL model as ground truth.

### Deep learning model

In our previous study [3], we generated the stacked convolutional layer and BiLSTM model for single-lead ECG delineation using the QT database. In the current study, we adjusted the model for the 12-lead ECG signal using LUDB. The convolution layers can extract deep features from ECG signal data points, and BiLSTM with forward and backward schemes help us learn from future and previous representations. We have trained the all beats from the start of P-wave_1_ to the start of P-wave_2_ for each lead (lead-by-lead). The rectified linear unit function (ReLU) was adopted with the convolution layer (8, 16, 32, 64, 128, 256, 512, 1024, and 2048 filters). We generated the 13 DL models using the convolution layers, LSTM and BiLSTM. Each fine-tuned hyperparameter of the 13 models is listed in Table 2. We fine-tuned—followed by applying varying convolution layers, from 1 to 4 with the LSTM classifier (Models 1–4) and 1 to 9 with the BiLSTM classifier (Models 5–13). All models were trained over 300 epochs, with a batch size of 8, a learning rate of 10^–5^, and categorical cross-entropy as the loss metric.

**Table 2:** Hyperparameter tuning of models

Model	Hyperparameters		
	Layer	Batch Size	Learning rate
1	Convolution 8 × 3, strides = 1 + ReLU - LSTM	8	10^–5^
2	Convolution 8 × 3, 16 × 3, strides = 1 + ReLU - LSTM		
3	Convolution 8 × 3, 16 × 3, 32 × 3, strides = 1 + ReLU - LSTM		
4	Convolution 8 × 3, 16 × 3, 32 × 3, 64 × 3, strides = 1 + ReLU - LSTM		
5	Convolution 8 × 3, strides = 1 + ReLU - BiLSTM		
6	Convolution 8 × 3, 16 × 3, strides = 1 + ReLU - BiLSTM		
7	Convolution 8 × 3, 16 × 3, 32 × 3, strides = 1 + ReLU - BiLSTM		
8	Convolution 8 × 3, 16 × 3, 32 × 3, 64 × 3, strides = 1 + ReLU - BiLSTM		
9	Convolution 8 × 3, 16 × 3, 32 × 3, 64 × 3, 128 × 3, strides = 1 + ReLU - BiLSTM		
10	Convolution 8 × 3, 16 × 3, 32 × 3, 64 × 3, 128 × 3, 256 × 3, strides = 1 + ReLU - BiLSTM		
11	Convolution 8 × 3, 16 × 3, 32 × 3, 64 × 3, 128 × 3, 256 × 3, 512 × 3, strides = 1 + ReLU - BiLSTM		
12	Convolution 8 × 3, 16 × 3, 32 × 3, 64 × 3, 128 × 3, 256 × 3, 512 × 3, 1024 × 3, strides = 1 + ReLU - BiLSTM		
13	Convolution 8 × 3, 16 × 3, 32 × 3, 64 × 3, 128 × 3, 256 × 3, 512 × 3, 1024 × 3, 2048 × 3, strides = 1 + ReLU - BiLSTM		

**Fig. 2:**
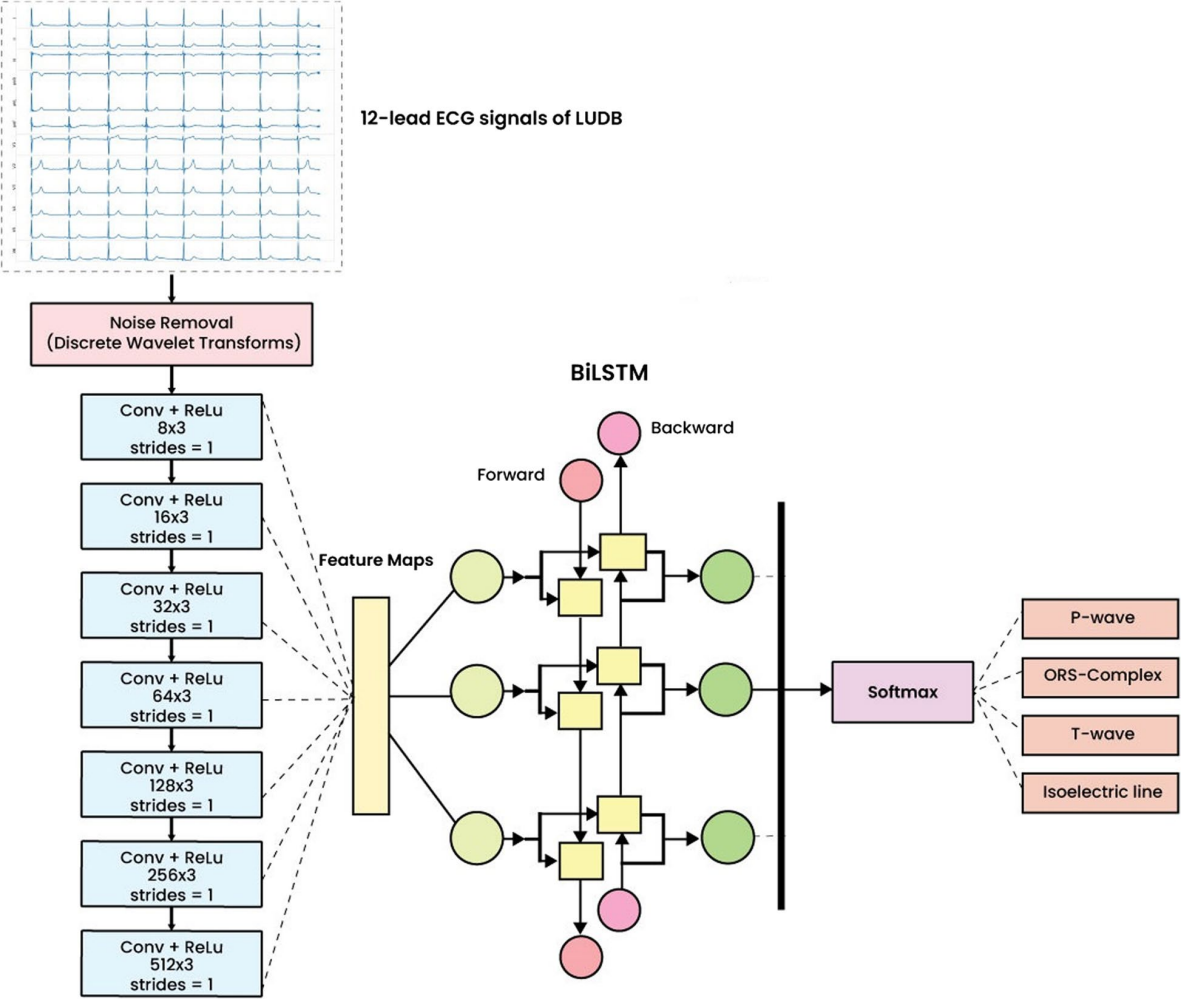
The proposed methodology of the 12-lead ECG delineation model

Among the hyperparameter-tuned models, the best model was Model 11. The pseudocode algorithm of the best model can be seen in Algorithm 1. The seven convolution layers and BiLSTM architecture were proposed as the ECG waveform classification model. The model takes the ECG signals as input and output multiclass classification (P-wave, QRS-complex, T-wave, and isoelectric line). The proposed network architecture is represented in Fig. 2. As shown in Fig. 2, the network consisted of seven convolution layers with varying filter numbers (8, 16, 32, 64, 128, 256, and 512 filters) and a kernel size of 3 to extract features. Convolution layers were used to automatically extract features and generate feature maps [33]. Feature maps were convolved by a trained kernel or filter and represent the intensity of ECG waveform features. A waveform with a similar intensity can be classified into the same class. Rectified linear unit layers performed nonlinear activation. The softmax function returns a probability of class membership for each class label, attempting to best approximate the predicted target for each input.

**Figure:**
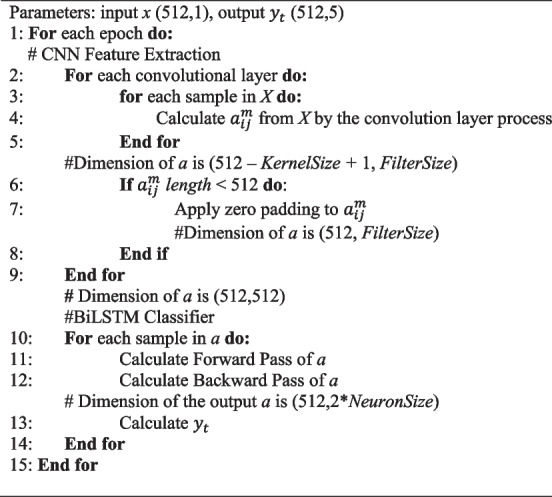
**Algorithm 1.** The pseudocode of CNN-BiLSTM

## Results and discussion

For this experiment, we segmented the waveform by beat-to-beat (start of P-wave_1_ to start of P-wave_2_) for each lead ECG. For the segmentation of beats, we have experimented based on two approaches: beat-based and patient-based. In this study, both approaches have be done to analyze the consistency of the performance set (training, validation and testing (unseen)) to avoid data leakage problems. The total of 200 records have randomly segmented (beat-to-beat segmentation). The total beat was 14,588 beats. For beat-based segmentation, the total of beats are consisting of 11,666 beats for training, 1,749 beats for validation, and 1,173 beats for testing (refer to Table 3). In addition, for patient-based segmentation, the total of beats are consisting of 13,331 beats for training, 645 beats for validation, and 621 beats for testing (refer to Table 4). We have used patients 1–180 as training set, patients 181–190 as validation set, and patients 191–200 as testing set. We have trained, validated and tested all beats for each lead (lead-by-lead).

**Table 3:** The total of P-wave_1_ to start of P-wave_2_ beat-based segmentation from 12-lead ECG signals

Data	Total number of beats segmentation											
	I	II	III	aVR	aVL	aVF	V1	V2	V3	V4	V5	V6
Training set	976	973	978	975	976	975	967	965	970	967	973	971
Validation set	147	146	147	146	146	146	145	145	145	145	146	145
Testing set	98	98	98	98	98	98	97	97	98	97	98	98
Total Beats	1221	1217	1223	1219	1220	1219	1209	1207	1213	1209	1217	1214

**Table 4:** The total of P-wave_1_ to start of P-wave_2_ patient-based segmentation from 12-lead ECG signals

Data	Total number of beats segmentation											
	I	II	III	aVR	aVL	aVF	V1	V2	V3	V4	V5	V6
Training set (patients 1 - 180)	1116	1112	1118	1114	1115	1114	1104	1102	1108	1104	1113	1111
Validation set (patients 181 – 190)	54	54	54	54	54	54	54	54	54	54	53	52
Testing set (patients 191 – 200)	51	51	51	51	51	51	51	51	51	51	51	51
Total Beats	1221	1217	1223	1219	1220	1219	1209	1207	1213	1209	1217	1214

The testing set was used as an unseen set because it showed model data that had never been seen before. The total number of beats segmentation is different due to only ground truth that provided the annotations waveform onset “(” and offset “)” are included. There are some unannotated waves. Also, some abnormal morphologies changes of ECG waveform, such as only upwards, only downwards, biphasic negative-positive, or biphasic positive-negative have affected the bias of total number of beats segmentation. For ECG waveform classification for 12-lead ECG signal, we used some performance metrics, such as accuracy (ACC), sensitivity (SEN), specificity (SPE), precision (PRE), and F1-Score (F1). As stated before, this study used 13 deep learning models for classification tasks.

### Beat-based segmentation

To generate the best model, we firstly experimented a beat-based segmentation approach and implemented the ACC, SEN, SPE, PRE, and F1 for the results of the 13 models (Table 5). Table 5 presents the performance results of 13 models with different hyperparameters. As shown in Table 5, the results of ACC, SEN, SPE, PRE, and F1 became higher as the convolution layers increased. LSTM was implemented in Models 1–4, and the highest ACC and PRE obtained were 98.05% and 93.06%, respectively. However, when the classifier was modified to BiLSTM, the highest results, 98.82% for ACC and 95.94% for PRE, were achieved (Model 11). The results seemed slightly insignificant when the eight and nine convolution layers were added (Models 12 and 13). Therefore, we proposed Model 11 as the best model for 12-lead ECG waveform classification in this study.

**Table 5:** The performance results of 13 hyperparameters tuning models (beat-based segmentation)

Model	Performance (%)				
	ACC	SEN	SPE	PRE	F1
1	97.43	90.99	98.29	90.95	90.95
2	97.77	91.80	98.51	91.96	91.85
3	97.92	92.11	98.60	92.63	92.34
4	98.05	92.73	98.70	93.06	92.86
5	98.23	93.64	98.80	93.98	93.80
6	98.31	94.01	98.86	94.20	94.10
7	98.47	94.66	98.98	94.69	94.67
8	98.59	95.11	99.06	95.06	95.07
9	98.70	95.33	99.12	95.56	95.44
10	98.78	95.65	99.18	95.84	95.74
11	98.82	95.93	99.21	95.94	95.93
12	98.74	95.63	99.16	95.52	95.56
13	98.70	95.33	99.12	95.56	95.44

**Fig. 3:**
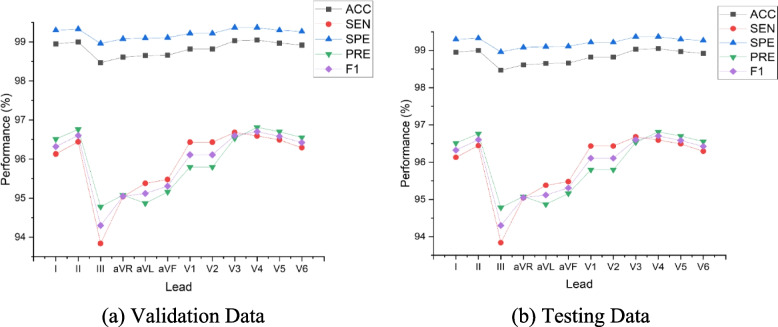
The performance results of 12-lead ECG from the best model to the validation and testing set (beat-based)

Figure 3 shows the 12-lead ECG performance results of Model 11 (the best model). As shown in Fig. 3a, we tested the best model with validation data (1749 beats) and found that the performance results of each lead showed no significant difference (above 93%). However, the highest precision and accuracy were achieved by the chest lead and lead V3 (99.03% and 96.53%, respectively). Also, lead V3 had good performance in all metrics. The good performance of lead V3 shows a nonspecific sign that a wide variety of ECG abnormalities, such as right bundle branch block due to inverted T-waves, could occur. Commonly, lead V3 is placed diagonally between leads V2 and V4. The ECG morphology of lead V3 observes the anterior wall of the left ventricle and is therefore named the anterior lead.

Furthermore, the worst performance result of the 12-lead ECG was lead III. The sensitivity and precision were only 93.84% and 94.78%, respectively. We had a challenge observing lead III, as its morphology was inverted almost the entire length of the ECG recording (refer to Fig. 1). Lead III kept track of the inferior aspect of the left ventricle. Figure 3b shows that model 11 was also tested using testing data (unseen). A total of 1173 beats were tested as unseen data. Like the results of the validation data, the testing data results showed no difference, and the performance result was above 93%. The best performance was also achieved by the chest lead (V3–V5), while the worst was lead III. Since the results of the chest lead outperformed the limb lead, this may reveal posterior ST-segment elevation myocardial infarction (STEMI).

**Fig. 4:**
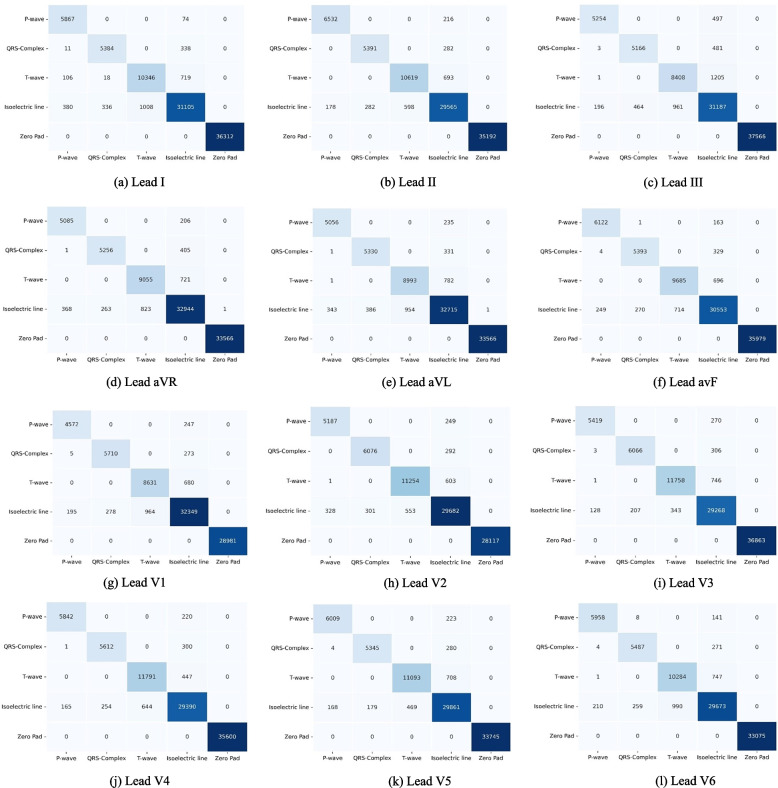
The confusion matrix of the best model in the ECG 12-lead performance

The confusion matrix (CM) has visualized to measure the performance of actual and predicted values (refer to Fig. 4). Each diagonal element of CM represents a successfully classified result. The off diagonals of the CM show the misclassified results. In light of this, the ideal classifier will have a CM with just diagonal members and all other values set to 0. Figure 4 shows the most misclassified occurs in isoelectric line, which falsely classified as P-wave, QRS-complex and T-wave and vice versa. However, among the P-wave and QRS-complex, the T-wave has the highest misclassified in all ECG 12-lead. The detection of T-wave is arduous due to the low amplitude, varying morphology (inverted, upwards, downwards, or biphasic T-wave) and its overlapping with P-wave. The minimal error of T-wave classification occurs mostly in lead I, II, and V2 – V5. T-wave represent the ventricular repolarization, and normal T-waves are upright in those leads.

**Fig. 5:**
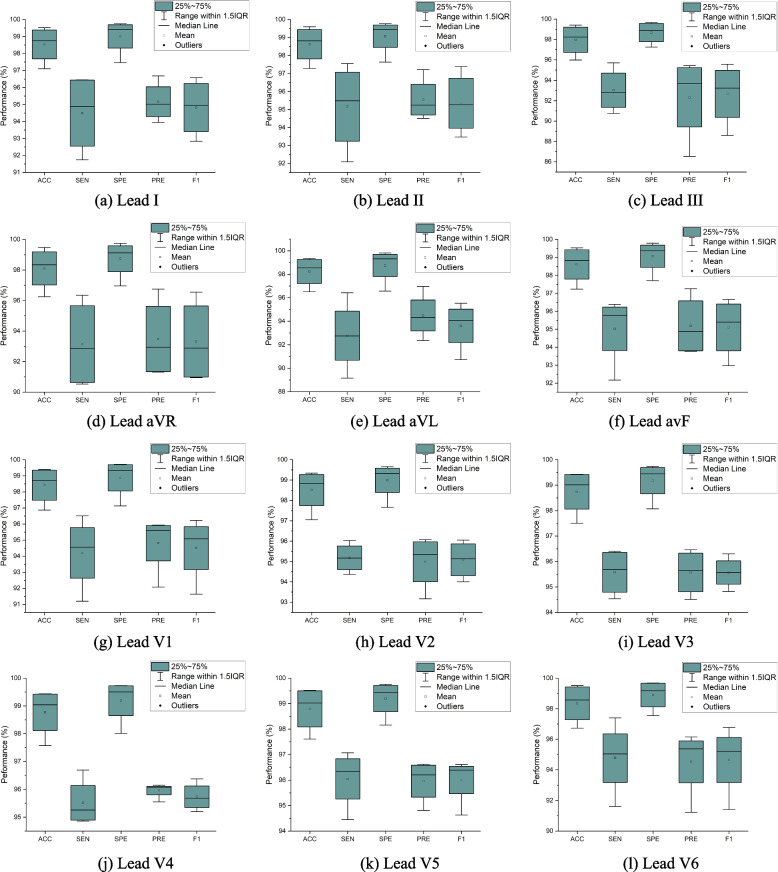
The boxplot of P-wave, QRS-complex, T-wave, and isoelectric line performance in 12-lead ECG

To present the performance results of each ECG waveform class (i.e., P-wave, QRS-complex, T-wave, and isoelectric line), we used the boxplot (also called a box and whisker plot) to display the distribution of the results based on the minimum, first quartile (Q1/25th percentile), median (the second quartile, Q2/50th percentile), third quartile (Q3/75th percentile), interquartile range (IQR), and maximum. Figure 5 shows that the values in the performance results were spread out. Leads I–III were achieved around a minimum of 86% PRE, and around 91% PRE in leads aVR, aVL, and avF. In chest leads (V1–V6), the performance changes were significant, with a minimum of 95% PRE. All boxplots of each lead do not suspect any anomalies due to errors in data collection. To reduce or remove outliers, the whisker on the appropriate side was drawn to 1.5 IQR rather than the data minimum or maximum.

**Fig. 6:**
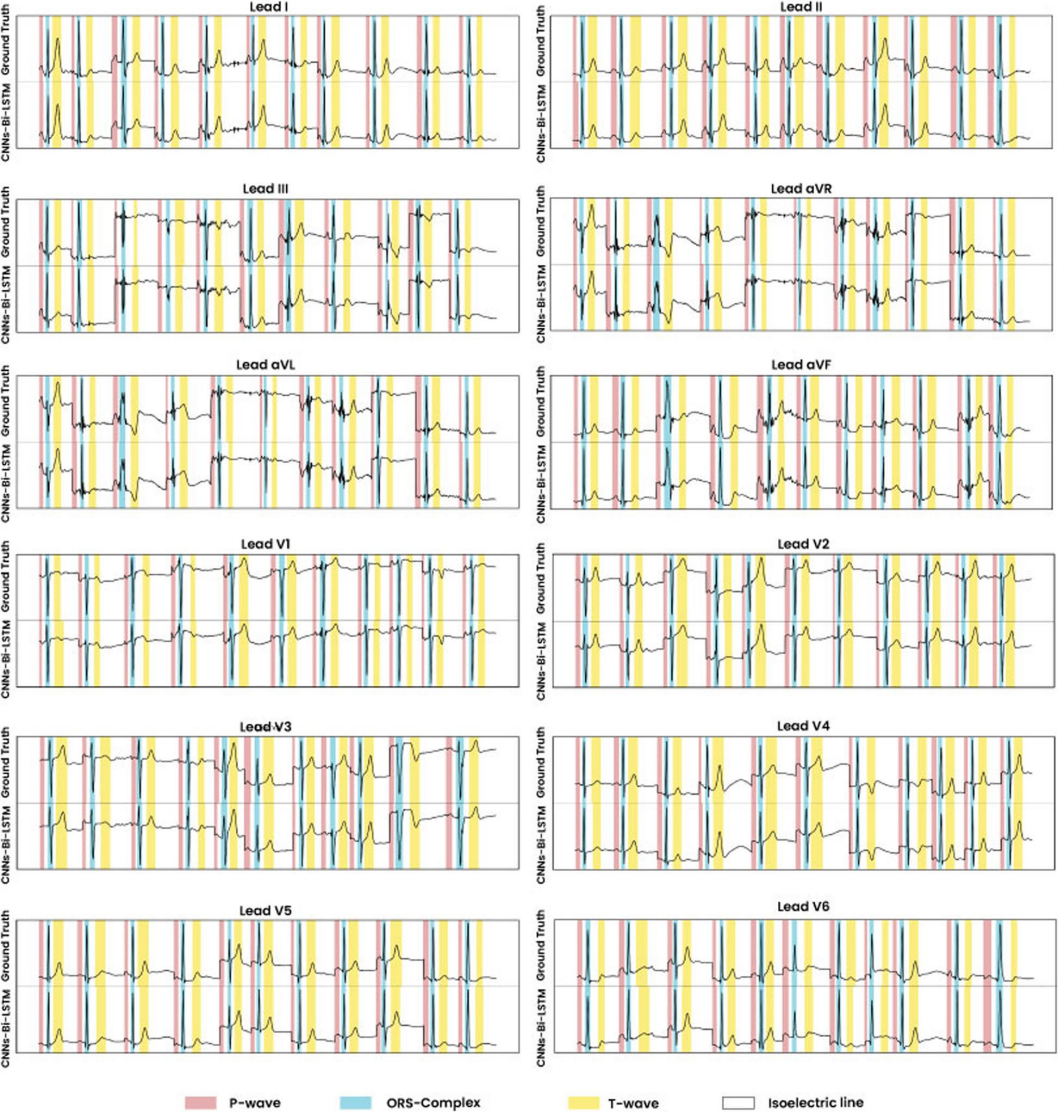
The comparison ECG waveform classification between ground truth and proposed CNN-BiLSTM model based on testing data (beat-based segmentation)

The result of the best model that shows the ground truth and the proposed CNN-BiLSTM model in testing data (unseen) is presented in Fig. 6. The ground truth is the ECG boundary of LUDB annotation from two cardiologists. Figure 6 shows that the error of delineation tends to be T-wave (yellow color) misclassified. T-wave represents ventricles repolarization and, basically, is difficult to detect, which is why a rather comprehensive discussion is needed. The P-wave (red color) indicates atrial depolarization and is mostly clear in lead II. In addition, QRS-complex (blue color) shows outstanding performance in almost all chest leads.

As seen in Fig. 6, the classification of ECG waveform boundary in 12-lead ECG is a challenging task. The morphological characteristic of each lead affects the delineation performance. Each lead represents the difference in electrical potentials measured at two points in space. Leads I, II, III, aVR, aVL, and aVF provide the 12 perspectives of the activity of the heart through the frontal plane, while the rest of the leads record the voltages of the heart onto the horizontal plane. The degree of morphological changes is determined by the ECG lead, the amount of the shift, the direction of displacement, and the ECG segment chosen for analysis.

### Patient-based segmentation

**Fig. 7:**
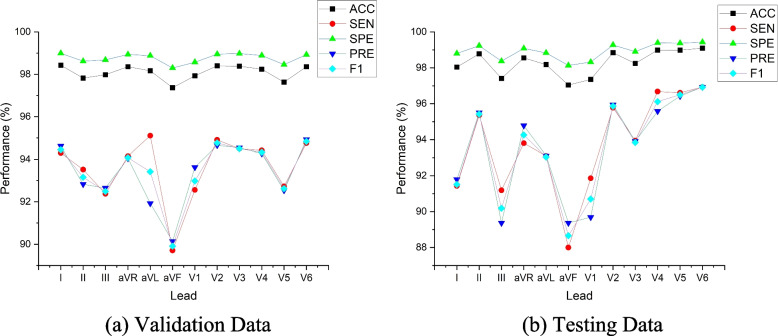
The performance results of 12-lead ECG from the best model to the validation and testing set (patient-based)

**Fig. 8:**
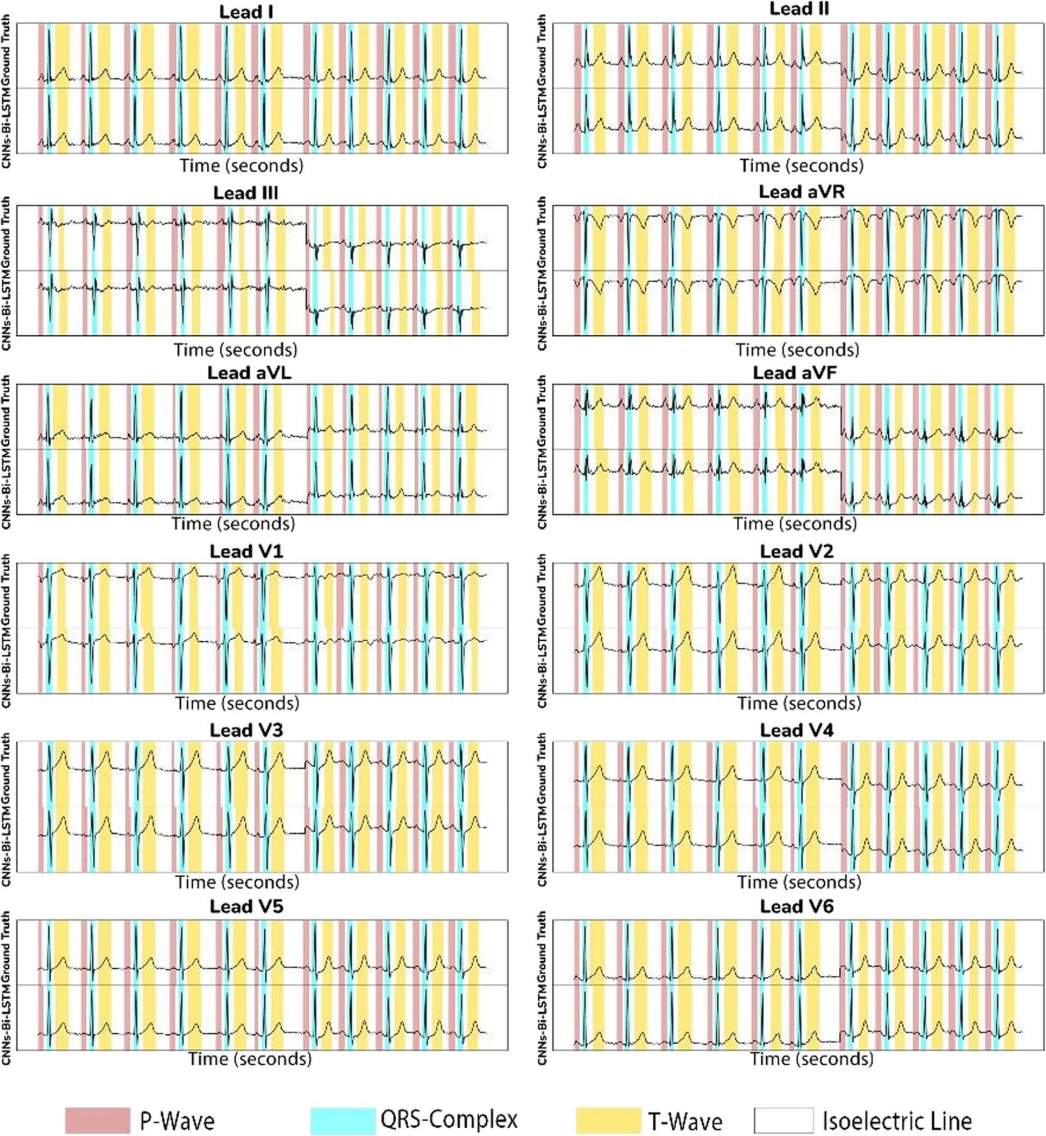
The comparison ECG waveform classification between ground truth and proposed CNN-BiLSTM model based on testing data (patient-based segmentation)

The hyperparameters tuning of Model 11 have retrained and experimented to a patient-based segmentation. With the same hyperparameters tuning, the performance results of patient-based segmentation can be presented in Fig. 7. Figure 7 presented the results of ACC, SEN, SPE, PRE and F1 from patient-based segmentation. Compared to the performance results of beat-based, beat-based segmentation outperformed patient-based segmentation even using the same hyperparameters. The average ACC of 12-lead achieved 93.72% for testing set, smaller than ACC of beat-based segmentation that was achieved 95.89%. Also, the performance results of SEN, SPE, PRE and F1 were decreased. In clinical practice, the characteristics of ECG signals in each patient are different. The varying ECG morphology of each patient can be considered, due to LUDB has varying heart rhythm types with related to heart disorders. The visualization of ECG waveform classification using testing set (unseen) can be presented in Fig. 8. Regardless of beat-based and patient-based segmentation, the performance results are well-performed with the ACC, SEN, SPE, PRE and F1 above 93%. We concluded both models can be implemented for delineation task. However, from the results comparison, the beat-based segmentation approach can be proposed in this study.

This investigation compares the proposed CNN-BiLSTM to other recurrent network algorithms, i.e., gated recurrent unit (GRU, bidirectional GRU (BiGRU) and unidirectional LSTM. They have been used as classifiers for ECG waveform classification in 12-lead ECG. The results are presented in Table 6. The varying results of all performance results are not significant; they ranged between 92.41% to 99.21%. However, the proposed BiLSTM classifier outperformed other recurrent network algorithms (GRU, BiGRU, and LSTM) for this investigation. In some cases, LSTM achieved more powerful results if compared to GRU though GRU has a simpler architecture with two gates (update and reset gates). It can be shown the proposed CNN-BiLSTM is worthy of to 12-lead ECG delineation task.

**Table 6:** The performance results of recurrent network algorithms

Algorithms	Performance Results (%)				
	ACC	SEN	SPE	PRE	F1
GRU	98.13	93.02	98.82	93.20	93.06
BiGRU	98.53	94.83	99.08	94.38	94.56
LSTM	98.03	92.41	98.75	93.20	92.75
BiLSTM	98.82	95.93	99.21	95.94	95.93

The proposed CNN-BiLSTM model also can be compared to other DL architectures as shown in Table 7. Previous studies have implemented a single-lead or multiple-leads to classify ECG waveform (i.e., P-wave, QRS-complex, and T-wave) using LUDB [34–37]. Some studies proposed U-Net architecture for automatic ECG delineation [34, 36]. Chen et al. [34] proposed U-Net architecture, which the encoder and decoder are being the main components of the proposed architecture. They have segmented the P, QRS, and T-waves using a single lead and achieved the sensitivities 99.88%. Moskalenko et al. [36] have also experimented U-Net architecture, which used two convolution layers and connected sequentially with MaxPooling layers. Their work used a lead II (single lead), and obtained the average of sensitivity and precision for the P, QRS, and T-waves are 99.23% and 98.99%, respectively.

**Table 7:** Comparison of the results of the delineation model to other DL architectures

Authors	Dataset	Lead	Class	Method	Performance Results (%)				
					ACC	SEN	SPE	PRE	F1
Chen et al. [34]	LUDB	1	4	U-Net	-	99.88	-	-	-
Liu et al. [35]	LUDB	1	3	ResNet and LSTM	-	99.83	-	-	-
Moskalenko et al. [36]	LUDB	1	3	U-Net	-	99.23	-	98.99	99.06
Jimenez-Perez et al. [37]	LUDB	Multi-lead	3	W-Net	-	99.93	-	99.87	-
**Proposed study**	**LUDB**	**12**	**4**	**CNN-BiLSTM**	**98.82**	**95.93**	**99.21**	**95.94**	**95.93**

Liu et al. [35] have proposed other architecture of CNN, ResNet, and hybrid to LSTM for P, QRS, and T-waves classification in a single lead. They experimented many kinds of ECG database, such as QTDB, LUDB, MITDB and BUT PDB. They used four convolution layers with the varying kernel size (4, 6, and 8). LSTM layer is connected to the residual network for achieving deep features. The proposed hybrid neural network has obtained the average of 99.83% sensitivity. Jimenez-Perez et al. [37] have explored the W-Net architecture, which it is the application of a second U-Net whose input is the output of the first U-Net. They have solved the issue of performance and complexity trade-off by using the efficient channel attention (ECA) mechanism. They experimented the ECG delineation task in single and multi-lead approach. For single lead, the precision of P, QRS, and T waves achieved 99.27%, 99.31% and 98.73%, respectively. However, when they applied the proposed model in multi-lead, the precision decreased to 98.90%, 99.24% and 98.24%, for P, QRS, and T-waves, respectively.

These works showed their proposed ECG delineation model is well-performed, however the scope of experimental study limited to the use of single lead ECG (mostly lead II). Also, in other perspective of the use of CNN in those studies, specifically for the convolution layers are excellent for feature extraction. Convolution layers may take advantage of any existing spatial and temporal patterns in the data.

Therefore, this study implemented the convolutional layers and BiLSTM to classify the 12-lead ECG waveform boundary. Also, it can be a preliminary task to develop and improve ECG delineation performance in cardiology clinical practice. The treatment of ECG signal processing in single and 12-lead ECG is different. The 12-lead ECG delineation is a more challenging task due to varying lead morphology. The repolarization and depolarization of ECG waveform can be arduous to handling. In this study, we have only experimented a single ECG database (LUDB), which has a single frequency sampling (FS). The proposed model has not been generalized to other 12-lead ECG databases due to the availability of data being limited. It can be our limitation for a preliminary task to generate the automatic 12-lead ECG delineation. The varying 12-lead ECG database can be explored for the future, with varying noise-handling technique and FS.

## Conclusion

The automatic delineation of the ECG main waveform in 12-lead ECG poses a challenge due to the characteristics of morphology appearance in each lead. By examining changes in ECG signal morphology, cardiologists can observe multiple heart disease processes. This study aimed to explore and improve the delineation model using the DL algorithm to classify the P-wave, QRS-complex, T-wave, and isoelectric line in a standard 12-lead ECG. This study generated the 13 hyperparameter tuning models and identified the best models using convolutional layers and BiLSTM. Also, for beat segmentation, we have experimented a beat-based and patient-based segmentation. The performance results were achieved above 95% ACC, SEN, SPE, PRE, and F1-score for beat-based segmentation. In addition, for patient-based segmentation approach, the performance results were achieved above 93% ACC, SEN, SPE, PRE, and F1-score. The performance results of beat-based segmentation outperformed the patient-based segmentation. Regardless of both performance results, any beat segmentation approach can be considered. The results can be proposed for a preliminary task to 12-lead ECG delineation. ECG examinations might be misinterpreted due to inaccuracies in electrode placement and variances in interindividual human anatomy. In the future, the unsupervised learning approach by training the network to remove ECG noise can be explored to replace the conventional wavelet transform. Also, the standard 12-lead ECG can be generalized for morphological changes diagnosis, not only limited to single-lead observation.

## Data Availability

The datasets generated and/or analysed during the current study are available in the PhysioNet:Lobachevsky University Electrocardiography Database repository, https://physionet.org/content/ludb/1.0.1/.

## Abbreviations

ACC: Accuracy
BiGRU: Bidirectional GRU
BiLSTM: Bidirectional long short-term memory
CNN: Convolutional neural network
DL: Deep learning
ECG: Electrocardiogram
F1: F1-Score
GRU: Gated recurrent unit
LA: Left arm
LL: Left foot
LUDB: Lobachevsky University database
LSTM: Long short-term memory
ML: Machine learning
PRE: Precision
ReLU: Rectified linear unit function
RA: Right arm
SEN: Sensitivity
SNR: Signal-to-noise ratio
SPE: Specificity
